# Innovations in immunotherapy for autoimmune diseases: recent breakthroughs and future directions

**DOI:** 10.3389/fimmu.2025.1647066

**Published:** 2025-09-17

**Authors:** May A. Alsayb

**Affiliations:** ^1^ Clinical Laboratory Sciences Department, College of Applied Medical Sciences, Taibah University, Madinah, Saudi Arabia; ^2^ Health and Life Research Center, Taibah University, Madinah, Saudi Arabia

**Keywords:** autoimmunity, immunotherapy, CAR T-cells, bispecific antibodies, checkpoint modulators, cytokine therapy, microbiome interventions

## Abstract

Millions of people worldwide suffer from chronic and devastating autoimmune disorders, challenging contemporary medicine. These disorders develop when the immune system attacks its own tissues, causing inflammation and damage. Traditional treatments have focused on widespread immunosuppression, which can relieve symptoms but has serious adverse effects and does not address immunological dysregulation. This review discusses the current and future trends in immunotherapy for the management of autoimmune diseases, including advancements such as CAR T-cell therapy, bispecific antibodies, next-generation immune checkpoint modulators, targeted cytokine therapies, and microbiome-based interventions. The discussion is grounded in current scientific literature, focusing on mechanisms of action, recent breakthroughs, limitations, and potential future directions. Each of the related sections presents cutting-edge advancements, current challenges, and future opportunities for research and clinical translation.

## 1 Introduction

Autoimmune diseases represent a significant challenge in modern medicine, being chronic and debilitating conditions affecting millions of individuals worldwide. These diseases occur when the immune system targets the body’s own tissues, leading to inflammation and tissue damage (1). Among these conditions, systemic lupus erythematosus (SLE), rheumatoid arthritis (RA), type 1 diabetes (T1D), and multiple sclerosis (MS) affect a global population of millions and pose a considerable challenge to treatment in terms of efficacy, safety, and long-term disease control (2).

Conventional therapy for autoimmune diseases has primarily focused on broad immunosuppression, which can alleviate symptoms but often has significant side effects and does not address the underlying immune dysregulation (3–5). More recently, immunotherapy has emerged as a revolutionary treatment approach. Unlike conventional therapies, immunotherapy aims to modulate the immune system more precisely by enhancing its regulatory functions or specifically targeting the pathogenic immune cells and molecules involved in the disease process (6, 7). This approach not only improves efficacy but also can reduce the adverse effects of the immune response. The impact of recent breakthroughs in immunotherapy for autoimmune diseases can extend beyond symptomatic relief, offering the potential for long-term disease remission and even cure. As research progresses, the focus on personalized medicine, combination therapies, and improved drug delivery systems continues to shape the landscape of autoimmune disease management (8). This evolving paradigm holds promise for transforming patient outcomes and improving the quality of life of those afflicted by these chronic conditions.

This review focuses on recent breakthroughs in immunotherapy for autoimmune diseases, including the adaptation of chimeric antigen receptor (CAR)-T cell therapy, the development of bispecific antibodies (bsAbs), advancements in next-generation checkpoint inhibitors, targeted cytokine therapies, and microbiome-based interventions. Each of the related sections presents cutting-edge advancements, current challenges, and future opportunities for research and clinical translation.

## 2 Chimeric antigen receptor T-Cell therapy in autoimmune diseases

**Figure 1:**
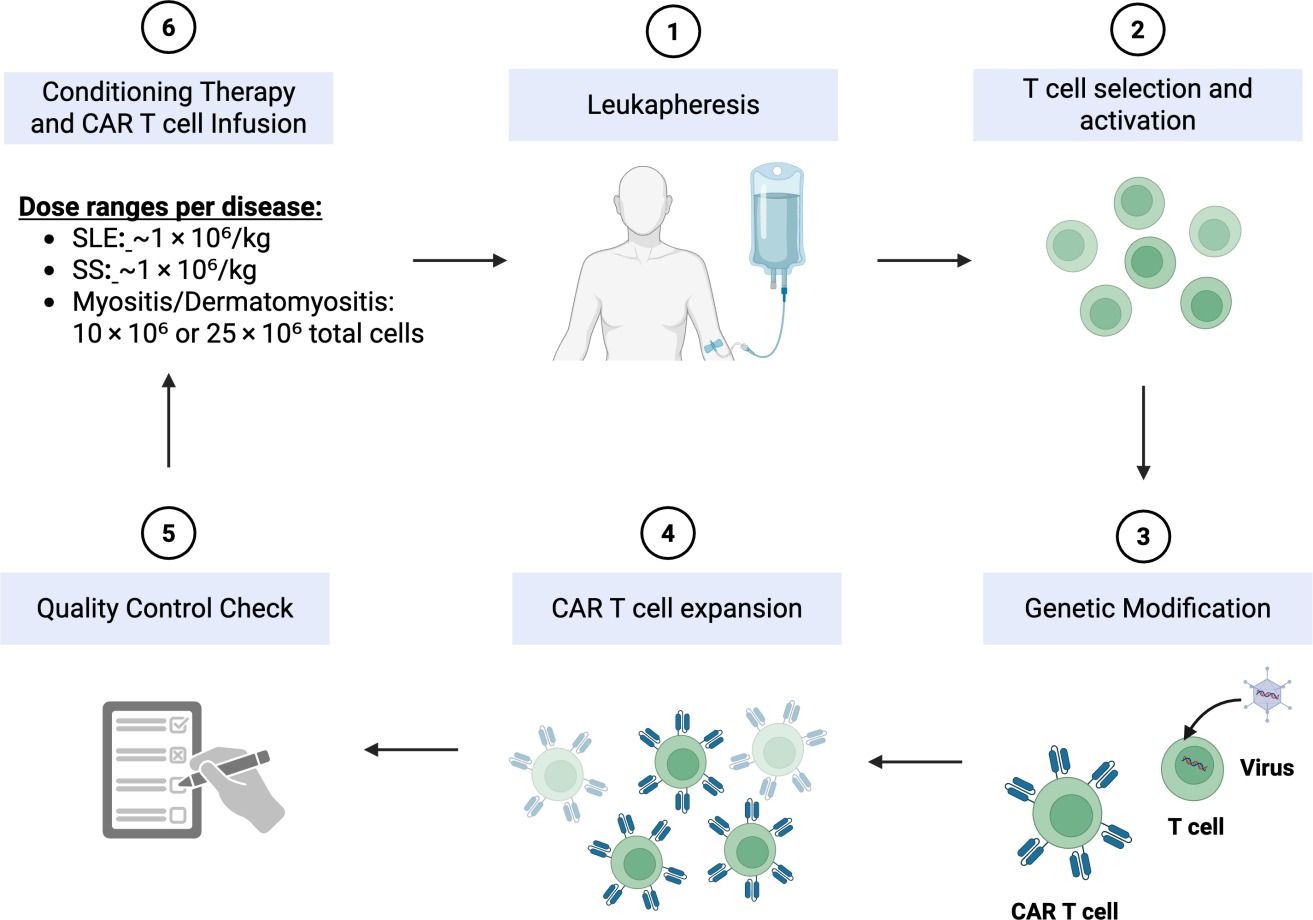
CAR T cell production model. Leukapheresis of the patient is the first step in producing CAR T cells, followed by T cell activation and enrichment. To help introduce and occasionally permanently integrate the CAR transgene, activated T cells are transduced (e.g., by a lentiviral vector). After expansion in either static or dynamic culture, gene-modified T cells are cryopreserved and reinfused into the patient. This figure is adapted from (10). Created in BioRender. Alsayb, M. (2025) https://BioRender.com/1glnsej.

CAR T-cell therapy has shown promise against treatment-resistant autoimmune diseases (9). By genetically modifying a patient’s autologous T cells to express synthetic receptors targeting specific antigens, CAR T-cell therapy allows for the selective elimination of autoreactive immune cells, thereby “resetting” immune tolerance (**Figure 1**). Schett et al. (7) demonstrated this transformative potential when they treated 5 patients with refractory SLE with CD19-directed CAR T cells. The results were remarkable: all patients entered durable drug-free remission, with normalized complement levels, decreased anti-dsDNA titers, and no further disease flares during follow-up. These results demonstrate the major role of autoreactive B cells in lupus pathogenesis and validate CAR T-mediated B-cell depletion as a possible disease-modifying intervention.

### 2.1 Breakthroughs

Recent studies show that CD19 CAR T-cell therapy can induce drug-free remission in refractory SLE and idiopathic inflammatory myopathies, with only mild, short-lived cytokine release syndrome as a well-tolerated side effect (11, 12). The therapy rapidly eliminates autoantibody-producing plasmablasts, and even after B-cell recovery, patients maintain remission with naïve, non-class-switched B cells over extended follow-up periods (13). Additionally, patients with systemic sclerosis experienced significant improvement in heart, joint, and skin manifestations, reinforcing the critical role of B-cell–mediated autoimmunity in these diseases and suggesting broader therapeutic potential for CD19 CAR T-cell therapy (14). Moreover, several preclinical and early clinical studies have looked into extending CAR T-cell therapy to other autoimmune conditions (15). Studies on CAR T-cell therapy for myasthenia gravis (MG) show that B-cell maturation antigen (BCMA)-targeted RNA-engineered CAR T cells led to clinical improvement and were deemed safe (16), while CD19-targeted CAR T cells achieved long-term disease stabilization without increasing infection risk, suggesting a safe and lasting treatment option for refractory MG (17, 18). Additionally, scientists at Xuzhou Medical University developed bispecific CAR T cells targeting CD19 and BCMA to reset immune responses in relapsed or treatment-resistant Chronic Inflammatory Demyelinating Polyneuropathy (CIDP), resulting in improved muscle function and reduced disability (19). Furthermore, CD19-directed CAR T cells ameliorated clinical disease in mouse models of MS and autoimmune encephalomyelitis, suggesting potential translational relevance for human autoimmune demyelinating diseases (20). Expanding the applications of CAR T-cell therapy, Lee et al. (21) demonstrated the use of desmoglein-3-specific CAR T cells in the preclinical treatment of pemphigus vulgaris, with the subsequent efficient elimination of autoreactive B cells. These milestones represent a paradigm shift: autoimmune disorders, previously treated only with lifelong immunosuppressants, may now be intervened upon, preferably through a single curative modality aimed specifically at the immunological cause (22). In essence, CAR T-cell therapy re-establishes the boundaries between oncology and immunology using precision immune-engineering. As for now, several clinical trials are currently ongoing, targeting different autoimmune diseases using CAR T-cell therapy (**Table 1**).

**Table 1:** CAR-T cell therapy currently in clinical trials for autoimmune diseases.

Clinical trial no.	Interventions	Conditions	Phases	Study status
NCT06279923	CD19-BAFF Targeted CAR T-cells	Autoimmune Diseases	Phase1	Recruiting
NCT05459870	4SCAR T cells	Autoimmune Diseases	Phase1, Phase2	Recruiting
NCT06688799	CD19 CAR-T cells	Autoimmune Diseases	Phase1, Phase2	Recruiting
NCT06685042	CAR T cell	Lupus Erythematosus, Systemic, System; Sclerosis, ANCA Associated Vasculitis, Dermatomyositis, Polymyositis	Phase1, Phase2	Recruiting
NCT06794008	BCMA-CD19 CAR-T therapy	Systemic Lupus Erythematosus, Inflammatory Myopathy, Systemic Sclerosis (SSc), ANCA-associated Vasculitis, IgG4-Related Diseases, Antiphospholipid Syndrome, Acquired Thrombotic Thrombocytopenic Purpura, Behcet Disease, Sjogren Syndrome	Phase2	Recruiting
NCT06435897	fetal MSCs combined with 4SCAR T cells	Autoimmune Diseases	Phase1, Phase2	Recruiting
NCT06428188	BCMA/CD19 CAR-T cells	Autoimmune Diseases, Systemic Lupus Erythematosus, Systemic Lupus Erythematosus Acute, Sjogren’s Syndrome	Phase1, Phase2	Recruiting
NCT06056921	CD19 targeted CAR-T cells	SLE (Systemic Lupus), Sjogren’s Syndrome, Systemic Scleroderma, Dermatomyositis, Anti-Neutrophil Cytoplasmic Antibody-Associated Vasculitis	Phase1	Recruiting
NCT06347718	anti-CD19 CAR T cell therapy	Systemic Lupus Erythematosus, Systemic Sclerosis, Dermatomyositis, Polymyositis	Phase1, Phase2	Recruiting
NCT06352281	CAR-T cells	ITP - Immune Thrombocytopenia	Phase1, Phase2	Recruiting
NCT06866080	LCAR-AIO T cells	Relapsed/Refractory Autoimmune Diseases	Phase1	Recruiting
NCT06941129	UCAR T-cell	SLE, Systemic Sclerosis (SSc), Inflammatory Myopathy, ANCA-Associated Vasculitis (AAV)	Phase1	Recruiting
NCT0637308	Anti-CD19-CD3E-CAR-T cells	Systemic Lupus Erythematosus (SLE), Sjogren’s Syndrome, Systemic Sclerosis, Inflammatory Myopathy, ANCA Associated Vasculitis, Antiphospholipid Syndrome	N/A	Recruiting
NCT06983964	CD19 CAR-T	Autoimmune Disease	N/A	Recruiting
NCT06249438	CD20/BCMA-directed CAR-T cells	Systemic Lupus Erythematosus (SLE), Immune-Mediated Necrotizing Myopathy, Neuromyelitis Optica Spectrum Disorders, Multiple Sclerosis-Relapsing-Remitting, Myasthenia Gravis	Phase1	Recruiting
NCT06231368	CNCT19 CAR-T cell therapy	Autoimmune Hemolytic Anemia, Autologous CD19 CAR-T, Failure of Three or More Lines of Therapy	Phase1	Active Not Recruiting
NCT06451159	KYV-101 anti-CD19 CAR-T cell therapy	Progressive Multiple Sclerosis	Phase1	Active Not Recruiting
NCT06350110	CD19- BCMA CAR-T cells	Systemic Lupus Erythematosus, Lupus Nephritis, Autoimmune Diseases, Anti-Neutrophil Cytoplasmic Antibody-Associated Vasculitis, Granulomatous Polyangiitis, Microscopic Polyangiitis, Systemic Sclerosis, Idiopathic Inflammatory Myopathies, Sjogren’s Syndrome	Phase1, Phase2	Recruiting
NCT06513429	IM19 CAR-T cells	Refractory Systemic Lupus Erythematosus	N/A	Recruiting
NCT06993493	CD19 CAR-T	Autoimmune Disease	N/A	Recruiting
NCT06822881	CAR-T Therapy	Systemic Lupus Erythematosus (SLE), Systemic Sclerosis (SSc)	Phase1	Recruiting
NCT06710717	CD19 CAR-T cells	Systemic Lupus Erythematous (SLE)	Phase1	Recruiting
NCT06775912	RD06–05 CAR-T Cell Injection	SLE, Systemic Sclerosis, IIM, NMOSD, MS, MG, ANCA Associated Vasculitis (AAV)	Phase1	Recruiting
NCT06869278	LCAR-AIO T cells	Multiple Sclerosis (MS), Neuromyelitis Optica Spectrum Disease (NMOSD), Anti-Myelin Oligodendrocyte Glycoprotein-IgG Associated Disorders (MOGAD), Myasthenia Gravis	Phase1	Recruiting
NCT06503224	Anti-BCMA and CD19 CAR-T cells will be injected intravenously on a one-time basis.	Autoimmune Diseases	N/A	Recruiting
NCT06904729	Low-dose CAR-T cells group, High-dose CAR-T cells group	Lupus Nephritis	Phase3	Recruiting
NCT06349343	CD19/BCMA CAR-T cell therapy	Systemic Lupus Erythematosus	Phase1	Recruiting
NCT06711146	CD19 CAR-T cells	Systemic Lupus Erythematosus	Phase1	Recruiting
NCT06549296	RD06–04 CAR-T Cell Injection	Systemic Lupus Erythematosus, Systemic Sclerosis, ANCA Associated Vasculitis, Idiopathic Inflammatory Myopathies, Sjogren’s Syndrome, Autoimmune Diseases	Phase1	Recruiting
NCT06548607	RD06–04 or RD06–05 CAR-T Cell Injection	SLE (Systemic Lupus), Systemic Sclerosis, ANCA Associated Vasculitis, Idiopathic Inflammatory Myopathies, Sjogren’s Syndrome, Autoimmune Diseases	Phase1	Recruiting
NCT06508346	anti-CD19-CAR-T cells	ANCA Associated Vasculitis, CAR-T Cell Therapy	N/A	Recruiting
NCT06361745	T cell injection targeting CD19 chimeric antigen receptor	Systemic Lupus Erythematosus, Idiopathic Inflammatory Myopathies, Systemic Sclerosis, IgG4 Related Disease, Primary Sjögren Syndrome	N/A	Recruiting
NCT06787989	BCMA-CD19 cCAR T cells	Refractory Immune Cytopenia	Phase1	Recruiting
NCT06138132	KYV-101 anti-CD19 CAR-T cell therapy	Multiple Sclerosis, Multiple Sclerosis, Primary Progressive, Multiple Sclerosis, Secondary Progressive	Phase1	Active Not Recruiting
NCT06733610	universal allogeneic anti-CD19/BCMA CAR T-cells	Autoimmune Hemolytic Anemia, CD19/BCMA CAR T-cells, Universal Allogeneic CAR T-cells	Phase1	Recruiting
NCT06934447	anti-BCMA/CD70-CAR-T cells	CAR T Cell Therapy, Systemic Lupus Erythematosus, BCMA	Phase1	Recruiting
NCT05938725	KYV-101 anti-CD19 CAR-T cell therapy	Lupus Nephritis, Lupus Nephritis - World Health Organization (WHO) Class III, Lupus Nephritis - WHO Class IV	Phase1, Phase2	Recruiting
NCT06222853	anti-CD19-CAR-T cells	Systemic Lupus Erythematosus, CAR-T Cell Therapy	Phase1	Recruiting
NCT06342960	KYV-101 anti-CD19 CAR-T cell therapy	Lupus Nephritis, Lupus Nephritis - WHO Class III, Lupus Nephritis - WHO Class IV	Phase1, Phase2	Recruiting
NCT04561557	CT103A cells, Cyclophosphamide and fludarabine	Autoimmune Diseases, Autoimmune Diseases of the Nervous System, Neuromyelitis Optica Spectrum Disorder, Myasthenia Gravis, Chronic Inflammatory Demyelinating Polyradiculoneuropathy, Idiopathic Inflammatory Myopathies, Multiple Sclerosis, Autoimmune Encephalitis, Myelin Oligodendrocyte Glycoprotein Antibody-Associated Disease (MOGAD), POEMS Syndrome	Phase1	Recruiting
NCT06920433	UCAR T-cell group	Systemic Lupus Erythematosus	Phase1	Recruiting
NCT06340750	LMY-920	Systemic Lupus Erythematosus	Phase1	Recruiting
NCT06212154	CAR-T19A	Autoimmune Hemolytic Anemia, CD19 CAR-T Cell Infusion	Phase1	Recruiting
NCT06626919	anitocel, Standard Lymphodepletion regimen	Muscular Diseases, Neuromuscular Manifestations, Autoimmune, Autoimmune Diseases, Autoimmune Diseases of the Nervous System, Myasthenia Gravis, Muscle Weakness	Phase1	Recruiting
NCT06585514	CD19 CAR-T cells	Systemic Lupus Erythematosus (SLE), Lupus Nephritis (LN)	Phase1, Phase2	Recruiting
NCT06465147	SCRI-CAR19v3	Systemic Lupus Erythematosus	Phase1	Recruiting
NCT05828212	CD19 CAR-T cells injection	Neuromyelitis Optica	Phase1	Recruiting
NCT06691152	CD19 Universal CAR-T cells	Systemic Lupus Erythematosus	Phase1	Recruiting
NCT06285279	FKC288	Lupus Nephritis, ANCA-associated Vasculitis, Membranous Nephropathy - PLA2R Induced, IgG4-Related Diseases	Phase1	Recruiting
NCT06947460	CD19-BCMA CAR-T cells infusion	Refractory Lupus Nephritis, Systemic Sclerosis, Primary Sjogren's Syndrome Combined With Pulmonary Hypertension	Phase1, Phase2	Recruiting
NCT06400303	KYV-101 anti-CD19 CAR-T cell therapy	Systemic Sclerosis, Systemic Sclerosis - Diffuse Cutaneous, Systemic Sclerosis - 2013 ACR/EULAR Classification Criteria	Phase1, Phase2	Recruiting
NCT06316791	single dose of CNCT19	Lupus Erythematosus, Systemic	Phase1	Recruiting
NCT05828225	CD19 CAR-T cells injection	Myasthenia Gravis	Phase1	Recruiting
NCT06294236	SC291	Lupus Erythematosus, Systemic Lupus Erythematosus, SLE (Systemic Lupus), Anti-Neutrophil Cytoplasmic Antibody-Associated Vasculitis, Granulomatous Polyangiitis, Microscopic Polyangiitis	Phase1	Recruiting
NCT06497361	PRG-2311	Lupus Nephritis, IgG4-related Disease	Phase1	Recruiting
NCT06193889	KYV-101, Standard lymphodepletion regimen	Myasthenia Gravis, Generalized Myasthenia Gravis	Phase2	Recruiting
NCT06371040	CD19-BCMA Targeted CAR-T Dose 1, CD19-BCMA Targeted CAR-T Dose 2, CD19-BCMA Targeted CAR-T Dose 2	Myasthenia Gravis	Phase1	Recruiting
NCT06384976	KYV-101, Standard lymphodepletion regimen, Anti-CD20 mAb	Multiple Sclerosis, Primary Progressive, Multiple Sclerosis, Secondary Progressive, Multiple Sclerosis, MS	Phase2	Active Not Recruiting
NCT06519565	PRG-1801	Immune Thrombocytopenia	Phase1	Recruiting
NCT06497387	PRG-1801	Lupus Nephritis, IgG4-related Disease	Phase1	Recruiting
NCT06785519	CD19/BCMA Lupus Nephritis Targeted CAR T-cells injection	Lupus Nephritis	Phase1	Recruiting
NCT06277427	PRG-1801 (CAR-T against BCMA)	Lupus Nephritis, ANCA Associated Vasculitis	N/A	Recruiting
NCT06653556	LCAR-AIO T cells	Systemic Lupus Erythematosus (SLE)	Phase1	Recruiting
NCT06797024	JY231 Injection	Autoimmune Diseases of the Nervous System	Na	Recruiting
NCT06544330	SYNCAR-001, STK-009	Systemic Lupus Erythematosus, Lupus Nephritis, Systemic Sclerosis	Phase1	Recruiting
NCT05988216	BRL-301	Systemic Lupus Erythematosus (SLE)	N/A	Recruiting
NCT06980597	OL-108	Systemic Lupus Erythematosus (SLE), Idiopathic Inflammatory Myopathy (IIM), Systemic Sclerosis (SSc), ANCA Associated Vasculitis (AAV)	Phase1	Recruiting
NCT06925542	CTX112	SLE (Systemic Lupus), Lupus Erythematosus, Systemic, Lupus Nephritis, Systemic Sclerosis, Inflammatory Myopathy, Idiopathic, Myositis, Diffuse Cutaneous Systemic Sclerosis	Phase1	Recruiting
NCT06588491	Standard lymphodepletion regimen	Stiff-Person Syndrome, SPS	Phase2	Recruiting
NCT06310811	RD06–04 Cells injection	Safety, Effective	N/A	Recruiting
NCT06462144	IMPT-514 CAR-T Cell Injection	Systemic Lupus Erythematosus (SLE), ANCA Associated Vasculitis (AAV), Idiopathic Inflammatory Myopathy (IIM)	Phase1	Recruiting
NCT06617793	rapcabtagene autoleucel (YTB323)	Relapsing Multiple Sclerosis	Phase1, Phase2	Recruiting
NCT04422912	DSG3-CAART or CABA-201	Pemphigus Vulgaris	Phase1	Recruiting
NCT06308978	FT819, Fludarabine, Cyclophosphamide, Bendamustine	Antineutrophilic Cytoplasmic Antibody (ANCA)- Associated Vasculitis (AAV), Idiopathic Inflammatory Myositis (IIM), Systemic Sclerosis (SSc), Systemic Lupus Erythematosus (SLE)	Phase1	Recruiting
NCT06121297	CABA-201	Systemic Lupus Erythematosus, Lupus Nephritis	Phase1, Phase2	Recruiting
NCT06359041	CABA-201	Generalized Myasthenia Gravis (gMG)	Phase1, Phase2	Recruiting
NCT06530849	GC012F Injection	Systemic Lupus Erythematosus	Phase1, Phase2	Recruiting
NCT06328777	CABA-201	Systemic Sclerosis, Scleroderma	Phase1, Phase2	Recruiting
NCT06799247	Decartes-08, OTHER: Placebo Drug	Myasthenia Gravis	Phase3	Recruiting
NCT06704269	GENETIC: YTB323	Generalized Myasthenia Gravis	Phase1, Phase2	Recruiting
NCT06902844	Equecabtagene Autoleucel Injection	Systemic Lupus Erythematosus (SLE), Lupus Nephritis (LN)	Na	Recruiting
NCT05451212	MuSK-CAART	MuSK Myasthenia Gravis	Phase1	Recruiting
NCT06897930	AZD0120, Cyclophosphamide, Fludarabine	Lupus Erythematosus, Systemic	Phase1, Phase2	Recruiting
NCT06333483	Obecabtagene autoleucel (obe-cel)	Systemic Lupus Erythematosus	Phase1	Recruiting
NCT06361836	SBT777101	Hidradenitis Suppurativa	Phase1	Recruiting
NCT06154252	CABA-201 following preconditioning with fludarabine and cyclophosphamide	Idiopathic Inflammatory Myopathy, Dermatomyositis, Anti-Synthetase Syndrome, Immune-Mediated Necrotizing Myopathy, Juvenile Dermatomyositis, Juvenile Polymyositis, Juvenile Idiopathic Inflammatory Myopathy (JIIM), Juvenile Myositis	Phase1, Phase2	Recruiting

Biomarkers linked to endothelial cell activation, such as the ANG2:ANG1 ratio (23) and soluble adhesion molecules (sVCAM-1, sICAM-1) (24), have also been associated with predicting CAR-T therapy response. A rise in these biomarkers often reflects a endothelial dysfunction, which is connected poor prognostic effect of CAR-T therapy (24, 25). An increase in IL-6 level is likewise associated with Cytokine Release Syndrome (CRS) induction, acting as a negative prognostic marker (26). Additionally, an increase in the immune exhaustion markers (PD-1, CTLA-4, LAG-3, TIM-3) affects T cell activities and predicts poorer CAR T cell therapy outcomes. Whereas, higher levels of cytotoxicity biomarkers like granzyme and perforin are linked to successful target cell death and a better outlook for CAR T cell therapy (25, 27). An increase in C-Reactive Protein (CRP) levels, a non-specific marker for systemic inflammation, is associated with severe CRS and may serve as an indicator of poor treatment outcome (25, 26, 28, 29). However, it’s essential to note that CRP is a non-specific inflammatory marker and its elevation can be associated with other conditions such as infection, trauma, or autoimmune activity. Thus, CRP alone without consideration of other clinical parameters cannot provide a conclusive interpretation. Moreover, the chemokines represent positive prognostic biomarkers, enhancing tumor targeting by increasing the CAR T cell homing to tumor sites (25, 30). Therefore, these biomarkers offer significant insights into the mechanisms affecting CAR T cell therapy outcomes, facilitating improved prediction of treatment efficacy and potential adverse effects, which is vital for optimizing patient management and enhancing prognoses (25).

### 2.2 Challenges

The widespread use of CAR T-cell therapy is impeded by major clinical and translational challenges despite recent advances. As with all therapeutic agents, CAR T-cell therapy entails some risk of adverse events like CRS, immune effector cell-associated neurotoxicity syndrome, and B-cell aplasia (31). The ratio of therapeutic benefit to over-immunosuppression remains finely poised in autoimmune disorders; this is important considering that the patient cohort may often already be immunocompromised (32). The logistical complexity of CAR T-cell therapy, which entails leukapheresis, ex vivo T-cell modification, expansion, and reinfusion, makes the therapy extremely costly and time-consuming, raising ethical and economic concerns regarding access (6). Furthermore, patient-specific characteristics such as baseline immune profiles, autoantibody specificity, or markers of T-cell exhaustion can have strikingly variable influences on treatment outcomes. Currently, these factors are poorly characterized, creating uncertainty in the clinic and complicating trial design.

### 2.3 Future directions

One potential avenue involves generating universal or “off-the-shelf” CAR T cells using CRISPR/Cas9 genome-editing techniques to create allogeneic T cells that lack endogenous T-cell receptors and Human Leukocyte Antigen (HLA) to prevent graft-versus-host disease and host rejection (33, 34). Furthermore, modifying naturally occurring regulatory T cells (Tregs) to express CARs with specificity for a given antigen is being investigated as a new approach in CAR therapy. This approach, currently under investigation for autoimmune conditions such as type 1 diabetes (T1D) and Crohn’s disease, aims not to eliminate immune targets but to suppress pathological immune activation and restore tolerance as a novel immunomodulatory strategy (35). To enhance safety, engineered “suicide switches” such as inducible caspase-9 (iCasp9) are incorporated into CAR constructs, allowing for rapid ablation of the therapy in the event of severe toxicity (36, 37). Moreover, machine learning algorithms are utilized to categorize patients according to predictive biomarkers of therapeutic response or adverse events. These models utilize multi-omics information, encompassing genomes, transcriptomics, and proteomics, to inform tailored therapy decisions (38–40). However, these new paradigms will not be validated until they demonstrate their value with regard to numerous, heterogeneous populations with longitudinal follow-up as well.

## 3 Bispecific antibodies for immune modulation

bsAbs are engineered molecules that can simultaneously engage 2 distinct targets, providing a unique mechanism of action that modulates immune responses more precisely than traditional monoclonal antibodies (41). This dual-targeting capability allows bsAbs to precisely target disease-relevant cells or pathways, reducing off-target effects and minimizing systemic immunosuppression. By engaging multiple targets, bsAbs can mitigate the development of resistance mechanisms that often compromise the effectiveness of single-target therapies (42, 43). This versatility of bsAbs can enable either immune suppression or immune redirection and thereby represents a major step forward in immunomodulatory therapy.

### 3.1 Breakthroughs

Several *in vivo* preclinical studies have demonstrated the potential of a bsAb designed to target both CD20 on B cells and CD3 on T cells in treating various autoimmune diseases, with promising results in terms of efficacy and safety (44, 45). This bsAb aims to deplete autoreactive B cells while simultaneously modulating T cell activity. The efficacy of Mosunetuzumab was assessed utilizing a humanized CD20/CD3 mouse model and an immune reconstitution model in NSG mice transplanted with human CD34^+^ cells. These studies have demonstrated the proof of concept for the application of CD20/CD3 bispecific antibody therapy in the treatment of autoimmune disorders. Mosunetuzumab exhibited a promising safety profile in SLE patients, characterized by only minor side effects and the absence of dose-limiting toxicities. Initial clinical findings, including as decreases in disease activity and autoantibody levels, together with B-cell depletion and T-cell activation, warrant further exploration of Mosunetuzumab as a prospective treatment for SLE (46). Moreover, Imvotamab, a CD20/CD3 bispecific antibody, is being explored for the treatment of refractory autoimmune diseases like RA and SLE due to its ability to deplete B cells with reduced cytokine release (45).

Furthermore, A study demonstrated the first use of blinatumomab, a bispecific anti-CD3/CD19 antibody, as a B-cell depletion therapy for systemic sclerosis, revealing profound B-cell depletion with no increase in infection risk, along with an improvement in clinical manifestations (47). However, this reflects a single case study, and the finding remains preliminary; thus, further investigations and studies should explore and confirm these findings.

bsAbs offer mechanistic advantages over monoclonal antibodies by enabling simultaneous modulation of multiple immune pathways (48). For example, a bispecific construct that targets TNF-α and IL-17A has been demonstrated to suppress both TNF-α and IL-17A, resulting in decreased inflammation in animal models, thus supporting its potential role as a therapeutic agent for RA (49). In a rheumatoid arthritis clinical trial, ABT-122, a bispecific antibody targeting TNF-α and IL-17, was shown to reduce the chemokines CXCL9, CXCL10, CCL23, and E-selectin, which are involved in the recruitment of T cells and/or myeloid cells, suggesting that ABT-122 may influence the trafficking of immune cell populations (50, 51).

In the context of T1D, a bsAb targeting both cytotoxic T-lymphocyte–associated protein-4 (CTLA-4) on T cells and Glucose Transporter Type 2 (GLUT2) on pancreatic β cells has shown potential in preserving β-cell function. By binding to GLUT2 on pancreatic cells and interacting with CTLA-4 on autoreactive T cell, it can promote tolerance and delay the onset of diabetes without apparent adverse effects, thus providing a potential therapeutic strategy for T1D (52). This dual-targeting strategy aims to modulate the autoimmune response specifically against β cells while promoting immune tolerance, offering a novel approach to modifying disease progression in T1D. Additionally, a current phase 2 clinical trial is evaluating the safety and efficacy of SAR442970 a bsAb targeting both TNF-α and OX40L in preserving pancreatic β-cell function in individuals recently diagnosed with T1D (NCT06812988). SAR442970 act by inhibiting TNF-α and OX40L, while TNF-α promote inflammation, OX40L interacts with the OX40 receptor on T cells, promoting their activation and survival. By blocking these two pathways, SAR442970 aims to reduce excessive immune activation and inflammation. Additionally, a limited number of other clinical trials are also in progress (**Table 2**).

**Table 2:** Bispecific antibodies currently in clinical trials for autoimmune diseases.

Clinical trial no.	Drug	Target	Conditions	Phases	Study status
NCT06900010	CM336	BCMA and CD3 Bispecific Antibody	Autoimmune Bullous Disease	Phase1 Phase2	Recruiting
NCT06647069	DR-0201	CD20 and Dectin-1 Bispecific Antibody	SLE (Systemic Lupus), CLE Cutaneous Lupus, Sjögren Syndrome, Primary Sjögren Syndrome, Dermatomyositis, Polymyositis Scleroderma, SSc, Diffuse Sclerosis, dcSSc, Diffuse Cutaneous Systemic Sclerosis	Phase1	Recruiting
NCT06975787	Vonsetamig	TNFRSF17/CD269 and CD3 Bispecific Antibody	Lupus Nephritis (LN)	Phase1	Not Yet Recruiting
NCT07010835	YK012	CD19 and CD3 Bispecific Antibody	Systemic Lupus Erythematosus (SLE)	Phase1 Phase2	Not Yet Recruiting
NCT06799611	CM336	BCMA and CD3 Bispecific Antibody	Immune Thrombocytopenia (ITP)	Phase2	Recruiting
NCT06181786	IMB-101	OX40L and TNF-α Bispecific Antibody	Rheumatoid Arthritis (RA)	Phase1	Not Yet Recruiting
NCT06982729	YK012	CD19 and CD3 Bispecific Antibody	Primary Membranous Nephropathy	Phase1	Recruiting
NCT07000292	MSC303	CD20 and CD3 Bispecific Antibody	Lupus Nephritis (LN), ANCA-Associated Glomerulonephritis	Phase1 Phase2	Not Yet Recruiting
NCT06812988	SAR442970	TNF-α and OX40L	Type 1 Diabetes Mellitus	Phase 2	Recruiting

### 3.2 Challenges

While bsAbs are designed to target CD3 and activate T cells toward autoreactive immune cells, uncoordinated CD3 engagement can overactivate T cells, resulting in excessive cytokine release (CRS) and tissue injury (53). It is noteworthy that while the most common adverse event associated with bsAbs is related to CRS, yet, it is typically milder than seen with CAR T-cell therapy (44). While early data on autoimmune conditions like SLE showed a favorable safety profile and temporary lymphocyte reduction, further studies are required to investigate the safety after long-term B cell depletion and its potential adverse events (44). Furthermore, safety and effectiveness of bsAbs depend on overcoming a number of significant challenges related to designing and manufacturing these bsAbs. For instance, the diverse molecular formats make manufacturing and purification more difficult, and their structural complexity lowers stability and raises the possibility of chain mispairing. It is also challenging to tune effector functions, ensure favorable pharmacokinetics, and achieve the proper binding balance for both targets (54). In addition, autoimmune diseases are massively heterogeneous; hence, the question of which pairs of antigens would form appropriate targets uniformly relevant across diverse populations of patients remains open. The lack of clear regulatory pathways for the use of bsAbs further adds to the complexities in introducing them into early human clinical studies. Despite the FDA’s guidance for bispecific antibodies in oncology, trial design is hampered by this regulatory gap, especially when it comes to defining safety margins, chronic dosing schedules, and patient comorbidities (55). Autoimmune patients often present with comorbidities and may be on concurrent immunosuppressive therapies. These factors complicate safety assessments and require tailored trial designs distinct from oncology protocols (42).

### 3.3 Future directions

Future innovation should be aimed at bsAbs that work on spatiotemporal control mechanisms for enhanced specificity and safety. With such approaches, conditionally active bsAbs may need inflammatory biomarkers or a particular tissue microenvironment to be activated (43). For example, pH-sensitive linkers or protease-activated formats are being researched to limit activity at the inflamed sites of joints or pancreatic islets (56). Such designs can provide safety against system toxicity as well as off-target effects for enhancement of the therapeutic index to a large extent. Furthermore, advancements in systems immunology and single-cell transcriptomics could allow for personalizing bsAb design according to patient immune signatures. Predictive models using artificial intelligence may better identify the possible antigen pairs for individual patients, thus paving the way for personalized bsAb therapy (57). Integrating these into modular bsAb platforms might allow for the fast adaptation of therapies for autoimmune conditions such as SLE, MS, and inflammatory bowel disease (IBD). There is limited follow-up data on safety, and the risk of immune activity, especially with non-human or chimeric domains, is still very much a concern (58). Future studies should investigate the establishment of biomarkers of durable tolerance induction and optimization criteria for dose regimens, as well as combinations with existing biologics or small molecules.

## 4 Next-generation checkpoint inhibitors

**Figure 2:**
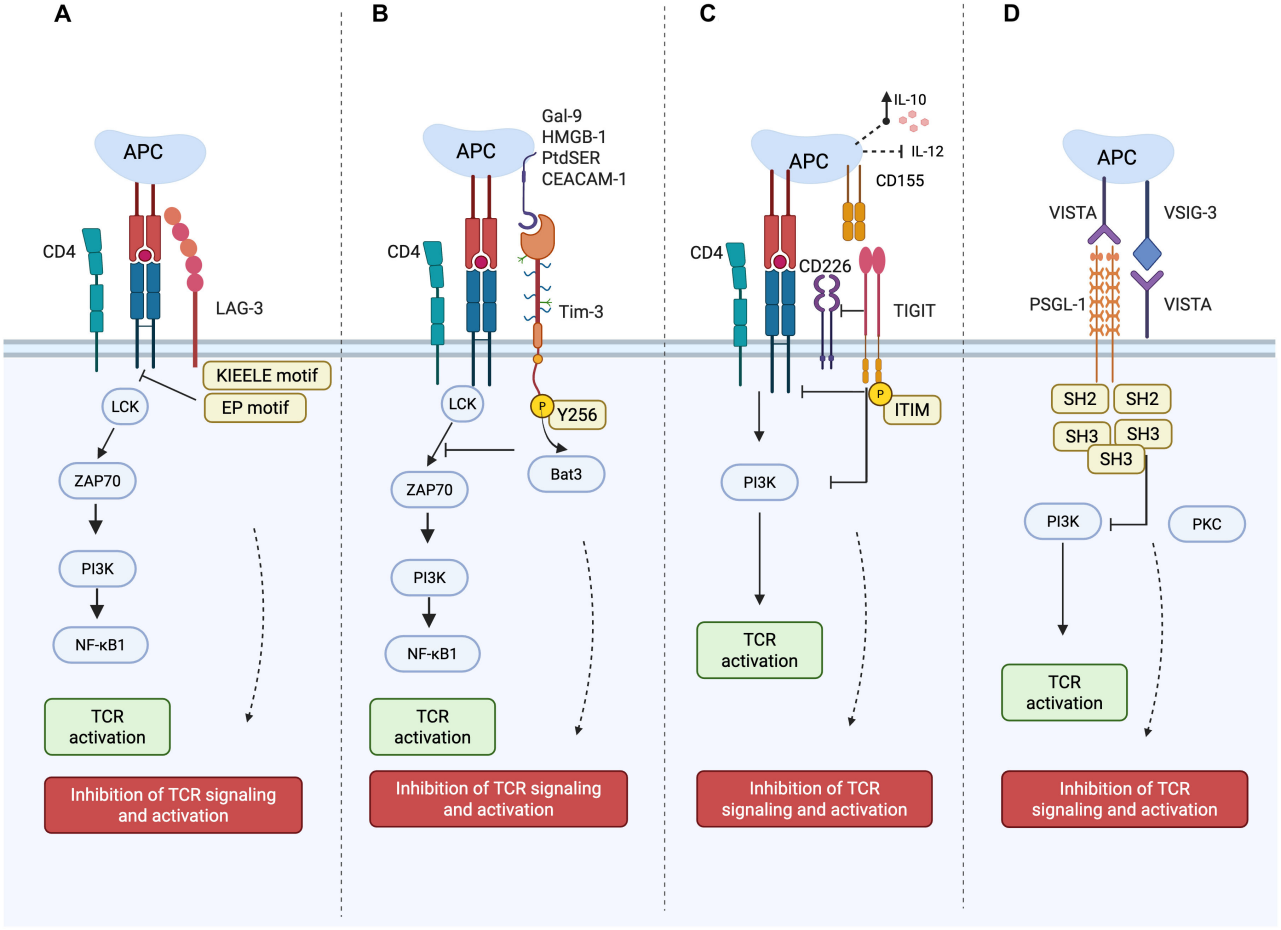
Checkpoint schematic pathway. Mechanisms of LAG-3, Tim-3, TIGIT, and VISTA inhibitory pathways in T cells: **(A)** Upon ligand binding, LAG-3 inhibits early steps of the TCR pathway in a manner dependent on LAG-3’s cytoplasmic domain. The KIEELE motif is responsible for regulating downstream inhibitory signaling, while EP motifs interfere with T cell activation by blocking CD3/Lck interactions. **(B)** TIM-3 binds to Gal-9 and induces phosphorylation at Tyr256 and Tyr263, releasing Bat3, which regulates Lck tyrosine kinases, thereby inhibiting TCR signaling. **(C)** In T cells, TIGIT exhibits a variety of inhibitory mechanisms. 1) TIGIT directly lowers TCR expression and TCR signaling by binding to CD155 and delivering intracellular inhibitory signals. 2) TIGIT can outcompete CD226 in CD155 binding because it binds to CD155 with a significantly higher affinity than its co-stimulatory counterpart CD226; 3) TIGIT interferes with CD226 homodimerization to prevent CD226-mediated T cell activation. 4) TIGIT inhibits T cells indirectly by binding to CD155 on APCs, thereby increasing IL-10 production and decreasing IL-12 production. **(D)** VISTA can function as a receptor that preserves a tolerant or quiescent phenotype and as a ligand expressed by tumor cells that inhibits T-cell receptor-mediated activation by signaling through a putative receptor on T cells. VISTA can bind to PSGL-1 in acidic environments and to VSIG-3 in physiological environments, inhibiting T cell function and proliferation. This figure is adapted from (62, 63). Created in BioRender. Alsayb, M. (2025) https://BioRender.com/r2iqbmx.

Inhibitory pathways, like programmed cell death-1 (PD-1), CTLA-4, and lymphocyte activation gene-3 (LAG-3), are crucial for maintaining the balance of tolerance to self and prevention of autoimmune pathology through the inhibition of overactive T cells (59, 60)., Immune checkpoint blockade reinvigorates exhausted T cells and enhances anti-tumor immune responses. In cancer, checkpoint inhibitors (e.g., anti–PD-1, anti–CTLA-4) block inhibitory receptors to unleash T cells against tumors. In autoimmunity, therapies may activate checkpoint pathways (e.g., CTLA-4 agonists like abatacept) to dampen overactive immune responses and restore homeostasis (61). Immune checkpoints such as LAG-3, T-cell immunoglobulin and mucin-domain containing-3 (Tim-3), T cell immunoglobulin and ITIM domain (TIGIT), and V-domain immunoglobulin suppressor of T cell activation (VISTA) are emerging targets for immune modulation and restoring immune tolerance and have been the focus of many studies on autoimmune disease (**Figure 2**).

### 4.1 Breakthroughs

LAG-3 is an immune checkpoint receptor that inhibits T-cell activation, proliferation, and contributes to immune homeostasis. In autoimmune-prone animal models, genetic deletion or pharmacologic blockade of LAG-3 exacerbates disease severity, underscoring its immunoregulatory role (64). Relapsing-remitting multiple sclerosis (RRMS) and T1D patients have a considerably low level of LAG-3^+^ CD4 and CD8 T cells. The low expression of LAG-3 has also been linked with T-cell resistance to apoptosis, promoting persistence of pathogenic T cells. These data suggest that LAG-3 agonists, by enhancing their inhibitory signaling, may be a promising target for restoring immune regulation in autoimmune conditions (65). Depending on the context of the disease, therapeutic approaches that target LAG-3 may involve either agonists or antagonists. Antagonists or depleting antibodies, like GSK2831781, are being studied to improve immune activity, while agonists may be investigated to suppress overactive immune responses in autoimmune diseases. GSK2831781, a monoclonal Ab targeting LAG-3 on activated T cells, can diminish LAG-3-expressing activated T cells in immuno-inflammatory conditions. Two clinical trials are assessing the safety and pharmacokinetics of GSK2831781 for the treatment of psoriasis (NCT03965533, NCT02195349). Another clinical trial was terminated in ulcerative colitis (NCT03893565) based on the assessment of clinical data, thus limiting the availability of this study’s safety and efficacy results. The preliminary data showed that GSK2831781 provides evidence of improvement in psoriasis; it has been shown to demonstrate the ability to downregulate the gene expression of *IL-17A*, *IL-17F*, *IFNγ*, and *S100A12* (66). Additionally, the LAG-3 agonist IMP761 is being investigated in a Phase I clinical trial (NCT06637865) involving healthy volunteers but has not yet been tested in autoimmune patients (multiple sclerosis). These different approaches, agonism versus depletion, show how complicated LAG-3 biology is and how important it is to have disease-specific strategies for autoimmune therapy.

Tim-3, an immune checkpoint receptor that is highly expressed on immune cells and induces immunological tolerance by suppressing T cell activation and promoting apoptosis. Tim-3 and MHC-II dysregulation are associated with MS and other autoimmune diseases. Mechanistically, Tim-3 suppresses MHC-II–mediated autoantigen presentation and CD4+ T-cell activation by downregulating MHC-II expression in macrophages via the STAT1/CIITA signaling axis. In murine models of experimental autoimmune encephalomyelitis (EAE), overexpression of Tim-3 reduced MHC-II levels and ameliorated disease severity, while its inhibition led to increased MHC-II expression and worsened clinical outcomes. These findings suggest that targeted modulation of the Tim-3 and MHC-II pathway, potentially through Tim-3 agonists, may offer a novel therapeutic strategy for restoring immune tolerance in MS (67). A recent study showed that LPX3, a liposomal formulation that targets Tim-3 and Tim-4, can trigger immune tolerance without the need for an antigen. LPX3 demonstrated its potential to restore immune regulation by effectively penetrating lymph nodes, colocalizing with immune cells, and promoting regulatory T cells expansion. According to these results, LPX3 might be a possible treatment approach for autoimmune disorders that does not require the co-administration of particular antigens (68).

TIGIT is a newly identified co-inhibitory receptor, that modulates the immune system. CD226 promotes positive signals, whereas TIGIT transmits negative signals, forming a route comparable to the CD28/CTLA-4 signaling pathway (69). TIGIT can induce immunological tolerance by suppressing autoreactive T cells, increasing tolerogenic dendritic cells (DCs), and encouraging the production and suppressive capacity of Tregs (70). Preclinical studies using a TIGIT-Ig fusion protein, agonist antibodies, and other modalities have demonstrated protective effects in murine models of autoimmune diseases, such as SLE (71) and experimental autoimmune encephalomyelitis (72). Beyond T and NK cells, TIGIT also influences regulatory B cells, which help dampen immune responses by inhibiting T cell activation and reducing pro-inflammatory DC activity (73). Although the role of TIGIT in B cells is less well characterized, emerging evidence suggests it may be central to autoimmune regulation. These findings position TIGIT as a promising candidate for immune checkpoint therapy in autoimmunity (73).

In a variety of autoimmune and inflammatory diseases, VISTA, a novel negative checkpoint receptor, functions as a negative immune regulator, thereby preventing excessive immune activation (74). In autoimmune conditions, such as lupus, MS, and RA, VISTA is essential for suppressing autoreactive T-cell responses, controlling monocyte and macrophage activation, and reducing the production of pro-inflammatory cytokines such as IL-17, IFN-γ, and IL-23. Research indicates that the deletion of VISTA increases inflammation, heightens immune cell infiltration, and exacerbates disease severity in the SLE animal model, hence affirming its protective function (75). In contrast, the activation of VISTA using agonist antibodies resulted in a reduction in inflammatory markers and an enhancement of clinical outcomes in many models, including lupus (76). The specific mechanisms differ among diseases; for instance, VISTA can modulate Th1/Th17 responses in MS and Toll-like receptor signaling in psoriasis. It has been reported that the use of VISTA blocking antibody in the EAE mouse model has increased the infiltration of IFN-γ^+^ and IL17A ^+^ producing CD4^+^ T cells in the central nervous system (CNS), exhibiting an activation of T cell-mediated immunity and loss of peripheral tolerance, thus increasing the susceptibility to EAE (77). On the Other side, in a model of psoriasis, VISTA-deficient dendritic cells are unable to adequately regulate the TLR7 pathway, which exacerbates IL-23/IL-17-driven skin inflammation (78). Therefore, its primary role is to preserve immunological equilibrium by regulating both adaptive and innate immune cells, thus underscoring its potential as a biomarker for immunological dysregulation. Despite these promising preclinical findings, no VISTA-targeted intervention has been authorized or progressed to clinical trials for autoimmune disorders, highlighting the promising opportunities for translational research to exploit its immunoregulatory capabilities (77).

### 4.2 Challenges

There are major obstacles to the therapeutic promise of next-generation checkpoint agonists. Of primary concern is the risk of extended immunosuppression, which may lead to opportunistic infections or malignancies in recipients (79). While preclinical models support efficacy, translating that efficacy to human autoimmune disease remains fraught with complexity due to heterogeneity in disease phenotype, complicating the prediction of patient responses and the identification of accurate biomarkers, such as TIGIT^+^ cell populations, for therapeutic monitoring (80). The dual functions of checkpoints, such as VISTA, which may be activated or downregulated according to the kind and stage of disease, complicate therapeutic targeting and necessitate meticulous, context-specific strategies (77). Moreover, although preclinical models have shown promising findings, the incomplete comprehension of the underlying mechanisms and off-target effects, as well as the potential for exacerbating immune dysregulation, limits the translation of these findings into effective and safe clinical therapies. Additionally, the interaction between co-inhibitory and co-stimulatory pathways (e.g., TIGIT/CD226) is complex and not entirely understood, complicating the development of medicines that optimize efficacy without inducing undesirable immune activation (73, 81). Furthermore, extended checkpoint activation may initiate the opposite of tolerance by exhausting Tregs or shifting antigen presentation dynamics, going against the original intent of therapy. Manufacturing agonistic antibodies with the correct affinity for binding, epitope specificity, and isotype to engage inhibitory receptors without inducing any off-target consequences further complicates the matter (82). Current biomarker panels also do not adequately predict patient outcomes, hampering the ability to personalize checkpoint-based therapies.

### 4.3 Future directions

Next-generation approaches, focusing on the tissue-restricted or cell-targeted delivery of checkpoint modulators, are being developed to tackle the above issues. Nanoparticle-based systems and antibody-drug conjugates are being evaluated for the localized delivery of PD-1 or CTLA-4 agonism to inflamed tissues such as pancreatic islets or synovial membranes to minimize systemic exposure (83). Synthetic biology is being investigated to develop checkpoint agonists engineered with logic gates to activate only in the presence of cytopathic cytokines or antigens. Another promising direction is the combination of immune repertoire sequencing with machine learning algorithms to stratify patients based on their chances of responding to checkpoint agonists. Subpopulations of autoreactive T cells that could display selective sensitivity to PD-1 or LAG-3 signaling might already have been distinguished through single-cell transcriptomics (84). Such predictive immune-profiling would allow for better selection of therapeutic targets with the mitigation of off-target immunosuppression. Despite current constraints, immune-checkpoint-modulating drugs appear to be an increasingly rational and viable new therapeutic option for autoimmune diseases. However, daunting empirical gaps concerning the expected long-term effects of chronic checkpoint engagement, durability of immune tolerance, and optimal design of checkpoint combinational therapy should be the focus of future research (81). Therefore, apart from proving efficacy, future clinical trials must generate robust biomarkers for safety and durability that allow personalized checkpoint immunomodulation.

## 5 Targeted cytokine therapies

Cytokines are considered key mediators in the immune system, regulating the recruitment, activation, and differentiation of various immune cells (85). Aberrant cytokine expression sometimes stimulates the persistent inflammation and tissue damage characterizing autoimmune diseases. Hence, a central strategy for treating autoimmune diseases has become targeting the specific cytokines modulating disease progression (86). While some of these therapies are already in clinical use, current research is further improving their specificity and enhancing their therapeutic reach.

### 5.1 Breakthroughs

Monoclonal antibodies targeting pro-inflammatory cytokines have revolutionized the treatment of autoimmune diseases, offering precision without broad immunosuppression. Among the most widely used are IL-6 inhibitors, such as tocilizumab and sarilumab, which have demonstrated clinical efficacy in rheumatoid arthritis (RA), systemic juvenile idiopathic arthritis, and giant cell arteritis (87). By blocking key drivers of autoimmune inflammation such as IL-6 signaling, these agents suppress acute-phase reactants (CRP, SAA, fibrinogen, etc), modulate B-cell activity, and inhibit Th17 cell differentiation (88). Building on this approach, IL-17A blockers (secukinumab, ixekizumab) and IL-23 inhibitors (guselkumab, risankizumab) have shown remarkable success in psoriasis, psoriatic arthritis, and ankylosing spondylitis. These cytokines are central to the Th17 axis, and their inhibition disrupts inflammatory circuits while preserving broader immune function (89). Monoclonal antibodies inhibit this axis and disrupt key inflammatory circuits without globally inhibiting the immune system. IFN-γ neutralization, although infrequently used, is beneficial in diseases such as hemophagocytic lymphohistiocytosis and has been investigated in autoimmune uveitis (**Box 1**) (90). In contrast to blocking inflammation, enhancing anti-inflammatory cytokines such as IL-10 represents a complementary strategy. While recombinant IL-10 has faced challenges due to poor pharmacokinetics and systemic toxicity, gene therapy and fusion protein delivery systems are being developed to enable localized, sustained release, potentially restoring immune balance without adverse effects (91, 92). Currently, several clinical trials are highlighting the translational progress of targeted cytokine therapy in autoimmune disorders (**Table 3**).

Box 1 **FN-γ neutralization in primary Hemophagocytic Lymphohistiocytosis (HLH).**

Primary Hemophagocytic Lymphohistiocytosis (HLH) results from a defect in cytotoxic T lymphocytes and natural killer cells' abilities to kill infected cells by perforin-mediated cytotoxicity. The uncontrolled activation of CTL and NK cells increases cytokine production that, in turn, hyperactivates macrophages and induces cytokine storm, where IFN-γ plays a particularly key role in the development of HLH. Emapalumab is a monoclonal antibody that functions by neutralizing IFN-γ by blocking its activity. It helps to reduce the excessive inflammation and immune activation associated with HLH. This therapeutic approach highlights the potential of IFN-γ inhibitors (e.g., Emapalumab) in managing macrophage activation syndrome (MAS), a severe complication of autoimmune diseases (89).

**Table 3:** Cytokine based therapy currently in clinical trials for autoimmune diseases.

Clinical trial no.	Intervention	Target	Conditions	Phases	Study status
NCT06663332	Guselkumab	IL-23	Crohn’s Disease, Ulcerative Colitis, Psoriatic Arthritis, Juvenile Arthritis	Phase3	Recruiting
NCT05083182	Ustekinumab Guselkumab	IL-12 IL-23	Juvenile Arthritis	Phase3	Recruiting
NCT06100744	Adalimumab Risankizumab	TNF-α IL-23	Juvenile Psoriatic Arthritis	Phase3	Recruiting
NCT06843239	Tibulizumab	BAFF IL-17	Systemic Sclerosis (SSc), Scleroderma	Phase2	Recruiting
NCT04589325	Ixekizumab	IL-17	Type 1 Diabetes Mellitus	Phase2	Recruiting
NCT06255028	CNTY-101, IL-2, Lymphodepleting Chemotherapy	CAR-iNK cell therapy, IL-2	Systemic Lupus Erythematosus, Lupus Nephritis, Idiopathic Inflammatory Myopathies, Diffuse Cutaneous Systemic Sclerosis	Phase1	Recruiting
NCT05339217	Telitacicept, IL-2	Inhibit the activity of two target cytokines (BAFF, APRIL)	Systemic Lupus Erythematosus	Phase3	Recruiting
NCT05631717	Human umbilical cord mesenchymal stem cells, IL-2	IL-2	Systemic Lupus Erythematosus, Lupus Nephritis	Phase3	Recruiting
NCT06544330	SYNCAR-001 STK-009	CD19-targeting CAR-T cell therapy, Orthogonal IL-2	Systemic Lupus Erythematosus, Lupus Nephritis, Systemic Sclerosis	Phase1	Recruiting
NCT05153070	Cyclosporin ILT101	IL-2	Type 1 Diabetes	Phase2	Recruiting
NCT06730126	Soquelitinib	inhibitor of IL2-inducible T-cell kinase (ITK).	Autoimmune Lymphoproliferative Syndrome	Phase2	Recruiting
NCT05428488	Abatacept (W12-W48), TNF Inhibitor (W12-W48), TNF Inhibitor (W0-W12)	blocking T-cell co-stimulation TNF inhibitor	Rheumatoid Arthritis	Phase3	Recruiting
NCT01793519	Etanercept, Infliximab, Adalimumab	TNF-α	Rheumatoid Arthritis	Phase4	Active Not Recruiting
NCT04378621	, TNF-α inhibitor OR JAK inhibitor,	TNF-α JAK inhibitor	Rheumatoid Arthritis, Pain, Fatigue, Cognitive Decline, Depression, Brain Diseases, Hand Rheumatism	N/A	Active Not Recruiting
NCT04870203	Baricitinib treatment, anti-TNF therapy,	JAK inhibitor. TNF-α	Rheumatoid Arthritis	Phase3	Recruiting
NCT06653634	Methotrexate, TNF Inhibitor	DHFR TNF inhibitor	Juvenile Idiopathic Arthritis	Phase4	Recruiting
NCT03227419	Tocilizumab Abatacept	IL-6 receptor (IL-6R) blocker. blocking T-cell co-stimulation	Rheumatoid Arthritis	Phase4	Recruiting
NCT06812988	SAR442970	Anti-CD40L monoclonal antibody	Type 1 Diabetes Mellitus	Phase2	Recruiting
NCT05306353	VIB4920 with TNFi, VIB4920 without TNFi	CD40L TNF	Rheumatoid Arthritis	Phase2	Recruiting
NCT05814627	Upadacitinib, Adalimumab,	Selective JAK1 inhibitor	Rheumatoid Arthritis	Phase3	Active Not Recruiting
NCT06440629	Therapeutic monitoring (TDM) of adalimumab		Rheumatoid Arthritis	Phase4	Recruiting
NCT06175338	Rituximab, MabThera^®^	CD20	Rheumatoid Arthritis	Phase1	Active Not Recruiting
NCT03976245	Etanercept, Tofacitinib	TNF-α JAK inhibitor	Rheumatoid Arthritis	Phase4	Recruiting
NCT03152058	Certolizumab Pegol	TNF-α	High Risk Pregnancy, Pregnancy Complications, Antiphospholipid Syndrome in Pregnancy, Lupus Anticoagulant Disorder	Phase2	Recruiting
NCT06100744	Adalimumab, Risankizumab	TNF-α IL-23	Juvenile Psoriatic Arthritis	Phase3	Recruiting
NCT02629159	Adalimumab, Upadacitinib	TNF-α JAK1 inhibitor.	Rheumatoid Arthritis	Phase3	Active Not Recruiting
NCT03414502	Methotrexate, Abatacept, Adalimumab, Azathioprine, Baricitinib, Certolizumab, Etanercept, Golimumab, Hydroxychloroquine, Infliximab, Leflunomide, Minocycline, Rituximab, Sarilumab, Sulfasalazine, Tofacitinib	DHFR CD80/CD86 TNF-α Purine synthesis JAK inhibitor TLR-7, TLR-9 DHODH CD20 IL-6R	Rheumatoid Arthritis	Phase3	Recruiting
NCT06527534	Filgotinib, Adalimumab	JAK1 inhibitor TNF-α	Rheumatoid Arthritis	Phase4	Recruiting
NCT04527380	Ixekizumab, Adalimumab	IL-17A TNF-α	Juvenile Psoriatic Arthritis, Enthesitis Related Arthritis	Phase3	Active Not Recruiting
NCT05305066	TNFi, Anti-IL6, JAKi	TNF-α IL-6 JAK	Rheumatoid Arthritis	N/A	Recruiting
NCT05626348	Iguratimod, Methotrexate, Adalimumab Injection, Leflunomide, Hydroxychloroquine	NF-κB DHFR TNF-α DHODH	Rheumatoid Arthritis	Phase4	Recruiting
NCT04909801	Abatacept, Adalimumab, Methotrexate	CD80/CD86 TNF-α DHFR	Rheumatoid Arthritis	Phase3	Active Not Recruiting

BAFF, B-cell activation factor; APRIL, A proliferation-inducing ligand; DHFR, dihydrofolate reductase; DHODH, dihydroorotate dehydrogenase.

### 5.2 Challenges

While cytokine-targeted therapies have proved effective, they come with significant downsides. A major reason is cytokine redundancy, wherein, since most cytokines have overlapping functions, blockade of a single cytokine leads to the compensatory upregulation of parallel pathways (93). For example, some IL-17 inhibitors result in disease activity reoccurrence in patients due to increased levels of IL-22, GM-CSF, or both, but all maintain inflammatory circuits independently. On the other hand, the long-term inhibition of cytokines leads to certain symptoms and effects caused by immunosuppression, such as increased susceptibility to bacterial, fungal, and viral infections (94). Patients treated with IL-6 blockade, for instance, may fail to show symptoms of an infection because fever and CRP production are suppressed, impeding diagnosis and treatment (95). Some cytokines may also have double functions depending on the tissue context; for example, IL-17 has a role in mucosal defense against several *Candida* species, and its inhibition is associated with mucocutaneous candidiasis (96). The diverse nature of autoimmune diseases means that cytokine profiles vary greatly across patients and even over time in the same patient. Therefore, applying the same set of single-cytokine blockers in every instance is not a valid approach, indicating that better tools for stratification, as well as dynamic biomarkers, are required (97).

### 5.3 Future directions

The above barriers have led to multifunctional approaches to next-generation cytokine therapies. Dual-cytokine inhibitors that target 2 cytokines synergistically are an interesting tool. An example is bimekizumab, which inhibits IL-17A and IL-17F, leading to superior efficacy over IL-17A blockade alone in psoriasis and spondyloarthropathies (98). Co-inhibition of IL-12 and IL-23 (e.g., ustekinumab) in Crohn’s disease and psoriasis modulates both Th1 and Th17 pathways. Another innovation aimed at enhancing specificity and reducing immunogenicity involves cytokine traps or engineered receptor decoys that sequester cytokines (99). Examples are rilonacept, an IL-1 trap used in autoinflammatory syndromes, and newer constructs targeting IL-6 and GM-CSF. Some cytokine traps are designed with altered Fc regions or joined ligands to enhance their half-life and improve tissue targeting (100). Synthetic receptors for cytokines under development can be engineered to switch pro-inflammatory signals into tolerogenic ones or restrict cytokine activity to defined tissues or cell types (101). For example, IL-2 variants engineered to preferentially expand Tregs while sparing effector T cells and NK cells are in late-stage clinical development for T1D and lupus (102). Advances in systems immunology and machine learning will play a transformative role in identifying cytokine signatures that predict therapeutic response. By integrating transcriptomics, proteomics, and spatial mapping of immune networks, cytokine therapy regimens can be tailored to each patient and their effects dynamically monitored.

## 6 Microbiome-based therapy

Microbiome-based therapy has recently become a promising approach in immunotherapy. The human gut microbiome is a major regulator of immune function. The microorganisms that populate the gastrointestinal tract greatly influence immune development, tolerance, and responsiveness (103). Imbalances in gut microbiota (dysbiosis) have been increasingly correlated with the etiology of several autoimmune diseases, including SLE (104), IBD, MS, T1D, and RA, leading an interest in microbiome-related therapies that seek to restore microbial balance and, accordingly, immune homeostasis (105). Dysbiosis has been shown to be correlated with the production of autoantibodies (106) and its effects are mediated by Th17/Treg balance, gut barrier integrity, and dietary interactions. While it is closely linked to B-cell differentiation, autoantibody production, and systemic immune responses in autoimmune diseases like SLE, RA, Graves’ disease, and Hashimoto’s thyroiditis, a direct cause-and-effect relationship is not firmly established across all conditions. Additionally, microbial metabolites, such as SCFAs, have been identified as important modulators of T-cell function and immune responses (107). Thus, microbiome-based immunotherapy has been extensively investigated for autoimmune disease treatment.

### 6.1 Breakthroughs

Probiotics and prebiotics are interventions designed to modulate immune responses through dietary measures. Preclinical studies that include animal models and *in vitro* studies demonstrated the ability of probiotics, including *Lactobacillus rhamnosus* GG, *Bifidobacterium longum*, and *Ruminococcaceae*, and *Lachnospiraceae*, to induce changes in the expansion of Tregs, reduce IL-6 and TNF-α as pro-inflammatory cytokines production, and restore mucosal barrier integrity (108, 109). What these measures imply is the influence that commensal microbes can have on systemic immune responses. In animal studies, dietary fibers (such as inulin and fructo-oligosaccharides) improved regulatory immunological responses (such as the growth of Tregs) and decreased disease severity. They also raised the number of beneficial SCFA-producing bacteria (110). Acetate and butyrate contribute to suppressing pro-inflammatory responses, while butyrate and propionate are especially crucial for promoting Treg differentiation through epigenetic regulation. These metabolites demonstrate how important gut microbial products are in regulating the immune balance of the host (111).

Fecal microbiota transplantation (FMT) now shows great promise in autoimmune contexts, particularly in ulcerative colitis (UC) (112). Multiple randomized controlled clinical trials have illustrated that FMT can induce clinical and endoscopic remission in patients with UC; the response rates correlated with increased microbial diversity, along with the enrichment of beneficial taxa, such as *Faecalibacterium prausnitzii* and *Akkermansia muciniphila* (113, 114). Preclinical studies investigating immune response following the transfer of healthy microbiota demonstrated a reduction in inflammation in RA and MS animal models. Moreover, FMT from healthy donors increased insulin sensitivity and delayed the onset of T1D in an animal model. A similar positive outcome was demonstrated with SLE, showing a decrease in autoantibody production and improvement in kidney function (115). Hence, several up-to-date clinical trials are actively investigating the therapeutic potential of the microbiome in the management of autoimmune diseases (**Table 4**).

**Table 4:** Microbiome**-**based therapy currently in clinical trials for autoimmune diseases.

Clinical trial no.	Intervention	Conditions	Phases	Study status
NCT04924270	FMT	Rheumatoid Arthritis, Ankylosing Spondylitis, Psoriatic Arthritis, Pulmonary Sarcoidosis, Crohn’s Disease, Ulcerative Colitis	Phase2	Recruiting
NCT03594487	FMT of FMP30 Donor Stool	Relapsing Remitting Multiple Sclerosis	Phase1	Active Not Recruiting
NCT05790356	FMT	Rheumatoid Arthritis, Fecal Microbiota Transplantation	N/A	Recruiting
NCT04096443	FMT	Multiple Sclerosis	Early Phase1	Active Not Recruiting
NCT06496412	FMT	Type 1 Diabetes	N/A	Recruiting
NCT04014413	FMT	Crohn’s Disease, Ulcerative Colitis, Celiac Disease, Irritable Bowel Syndrome, Functional Dysphonia, Constipation, Clostridium Difficile Infection, Diabetes Mellitus, Obesity, Multi Resistant Infection, Hepatic Encephalopathy, Multiple Sclerosis, Pseudo-Obstruction, Carbapenem Resistant Enterobacteriaceae Infection, Vancomycin Resistant Enterococci Infection, Multiple Organ Dysfunction Syndrome, Dysbiotic Bowel Syndrome, MRSA Enteritis, Pseudomembranous Enterocolitis, Alopecia, Autism, Graft-versus-host Disease, Idiopathic Thrombocytopenic Purpura, Atopy or Allergy, Liver Disease, Alcohol Dependence, Psoriatic Arthropathy	N/A	Recruiting
NCT07083882	Freeze dried autologous encapsulated FMT	Type 1 Diabetes (T1D), Fecal Microbiota Therapy (FMT)	Phase2	Recruiting

FMT, Fecal microbial transplant.

### 6.2 Challenges

Microbiome-based therapies face several major obstacles. The predominant obstacle constitutes individual variation, wherein every person has a unique microbial fingerprint shaped by genetics, environment, diet, and antibiotic use. Thus, some therapies may work for an individual or cohort but be useless, or worse, harmful, to another (116). Furthermore, FMT lacks standardization on donor selection, preparation, administration routes, and dosing frequency. This heterogeneity hampers reproducibility and raises safety concerns, like the potential transfer of opportunistic pathogens or undesirable metabolic characteristics (117, 118). Although screening protocols have improved, rare but severe adverse events, as well as the transmission of multidrug-resistant organisms, have triggered the FDA to intensify its controls. The mechanistic basis of microbiome-immune interactions is complex and not completely appreciated. Microbes affect immunity through different means, such as short-chain fatty acid production, changes in antigen-presenting cells, modulation of epithelial tight junctions, and interactions with pattern-recognition receptors (119). This issue is rendered even more complicated by the dynamic nature of the microbiome, which hinders persistence over time. Changes in diet, stress, sickness, or antibiotic consumption can drive a drastic shift in the community, compromising or entirely reversing previous benefits (120, 121).

### 6.3 Future directions

The future of microbiome therapeutics is in precision modulation interventions targeted to the individual’s specific microbial and immunological landscape. Next-generation sequencing and metagenomic profiling allow for the deep characterization of microbial communities and their functional potential and the identification of dysbiosis biomarkers and predictive response signatures (122). Currently, with the help of CRISPR-Cas systems, the idea is to edit bacterial genomes in the gut, thus allowing *in situ* reprogramming of the microbiome without the need to introduce foreign microbes into the environment. CRISPR-guided antimicrobials can kill pathogenic and pro-inflammatory strains while leaving beneficial commensals intact. However, gene insertion may enable the expression of immunostimulatory molecules by endogenous microorganisms (123). Synthetic biology is also accelerating the development of LBPs for sensing environmental cues and the controlled, tissue-specific release of therapeutic agents. Smart probiotics engineered to detect inflammation and respond by producing IL-10 or retinoic acid are part of this picture. Finally, computational modeling and machine learning are applied to simulate host-microbe interactions and predict treatment responses (124). By integrating all microbiome-initiated datasets on the genetic, dietary, and immune fronts, these tools will allow for the rational design of patient-specific microbiome therapies with optimized efficacy and safety.

## 7 Conclusion

**Figure 3:**
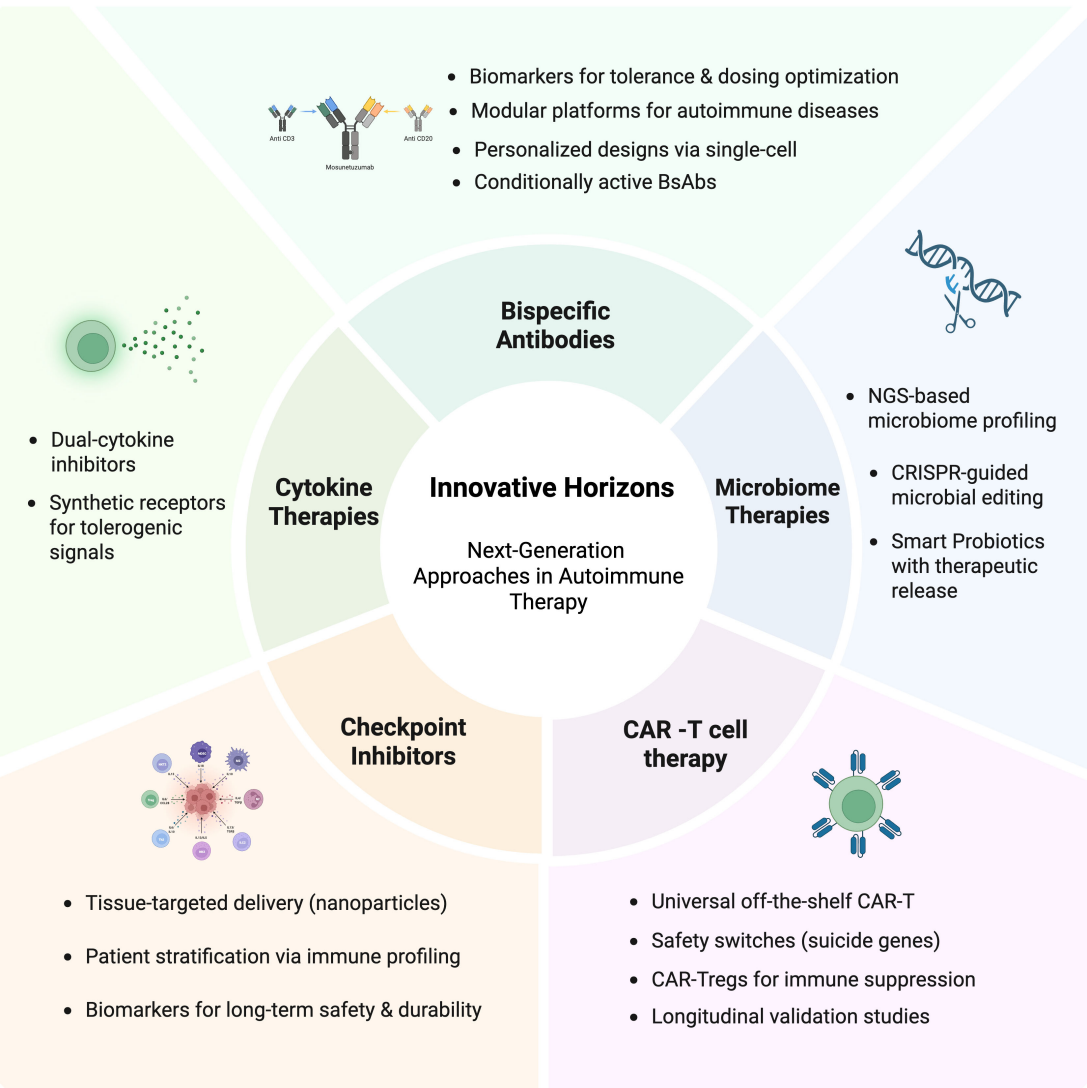
Innovative Horizons in Next-Generation Therapeutic Approaches for Autoimmune Diseases. This figure demonstrates the emerging schemes directed at immune modulation. These include CAR T-cell therapy, bispecific antibodies, next-generation immune checkpoint modulators, targeted cytokine therapies, and microbiome-based interventions, representing the foreground of therapeutic advancement in autoimmunity. Created in BioRender. Alsayb, M. (2025) https://BioRender.com/o15madg.

Immunotherapy for autoimmune diseases is undergoing a transition, shifting from immunosuppression to immune modulation. This trend can be observed in the advanced fields of CAR T-cell therapy, bsAbs, checkpoint agonists, cytokine targeting, and microbiome interventions, which reflect a new system of rapid change in therapeutics. Each has distinct advantages but presents challenges to safety, cost, scale, or regulatory approval. The future integration of such therapies into certified care as they advance in preclinical and clinical studies will depend on interdisciplinary research, technological refinements, and personalized approaches concerning patients’ various immune profiles (**Figure 3**). The future of autoimmune disease treatment will hinge on a personalized, multifaceted approach that incorporates immune modulation, tolerance establishment, and tissue regeneration.
